# Strain measurement with multiplexed FBG sensor arrays: An experimental investigation

**DOI:** 10.1016/j.heliyon.2023.e18652

**Published:** 2023-07-28

**Authors:** Bruno da Silva Falcão, Ausama Giwelli, Melissa Nogueira Kiewiet, Stephen Banks, George Yabesh, Lionel Esteban, Leigh Kiewiet, Nurudeen Yekeen, Yevhen Kovalyshen, Ludwig Monmusson, Ahmed Al-Yaseri, Alireza Keshavarz, Stefan Iglauer

**Affiliations:** a School of Engineering, Edith Cowan University, Joondalup WA 6027, Australia; b CSIRO Energy, Kensington WA 6151, Australia; c Currently at Fluid Science and Resources Division, Department of Chemical Engineering, University of Western Australia, Crawley WA, 6009, Australia; d Currently at INPEX, Perth WA 6000, Australia; e CSIRO Energy, Clayton VIC 3168, Australia; f Currently at Centre of Integrative Petroleum Research (CIPR), College of Petroleum Engineering and Geoscience, King Fahd University of Petroleum and Minerals, Saudi Arabia

**Keywords:** FBG sensor, Unconfined compressive strength (UCS), Hydrostatic test

## Abstract

In conventional rock mechanics testing, radial strain measuring devices are usually attached to the sample's surface at its mid-height. Although this procedure provides a realistic picture of the lateral deformation undergone by homogeneous samples, however, this assumption may not be accurate if the tested rock has significant heterogeneity. Fibre Bragg Grating (FBG) sensors have recently been introduced to various rock testing applications due to their versatility over conventional strain gauges and radial cantilevers. FBG sensors have small size, multiplexing capability, and immunity to magnetic interference. The main objective of this study is to explore and understand the capabilities of FBG sensing for strain measurement during rock mechanics testing, including under confining. To do so, two limestone plugs (Savonnières limestone) and one acrylic Poly Methyl Methacrylate (PMMA) plug, all of 38 mm diameter, were prepared. The acrylic plug and one of the Savonnières samples plugs were subjected to Unconfined Compressive Strength (UCS) tests. The second Savonnières plug was subjected to a hydrostatic test up to 20 MPa confining at room temperature. FBG sensors of 125 μm cladding diameter with ceramics (Ormocer) coating were glued on the surface of each sample, spreading across the entire sample's height. Strain gauges and cantilever-type radial gauges were used on the samples submitted to UCS for comparison. Results show that radial strain measurements and calculated elastic properties derived from the FBG readings for samples are comparable to readings from the conventional strain gauges and cantilever-type devices. Apparent bulk moduli based on volumetric strain computed from FBG radial strain readings during the hydrostatic test on the Savonnières sample was consistent with benchtop measurements conducted on the Savonnières sample and another plug extracted from the same parental block, as well as published literature data. Moreover, variations in the calculated elastic properties are interpreted as evidence that the FBG sensors detected heterogeneities in the samples' inner structure, which can be seen in the density profiles computed from x-ray CT images. Such observation confirms the potential of the presented FBG sensors configuration for 3D strain mapping in rock mechanics tests.

## 1 Introduction

A typical rock mechanics laboratory experiment involves the application of stresses on a cylindrical rock sample under well-controlled conditions while measuring the resulting strain response. The rock deformation is usually recorded via strain gauges, LVDTs (Linear Variable Differential Transformers) or cantilever systems to physically measure any compression and distension experienced by the sample during the test [1]. These devices are either glued or attached to the sample's lateral surface, either directly or over the testing sleeve, at specific punctual locations, often towards the middle height of the sample.

In homogenous materials, it is commonly accepted that these measurements represent the maximum radial (or axial) strain experienced by the material due to the imposed load and that this response portrays the uniform deformation undergone by the sample. However, for heterogeneous materials, a couple of mid-height strain measurements may have limited ability to fully depict the rock strain response as they may not capture the actual strain undergone by different sections of the sample. Furthermore, boundary effects such as endcaps across the length of the tested sample, slippage and compliance of sleeve/Viton jacket, could impact the accuracy of strain measurement [2]. As geomechanics laboratory results are often used to estimate or calibrate the mechanical response of rock formations on a field scale, it is important to improve the accuracy of laboratory-scale strain data, especially if heterogeneity is known to play an important role, e.g., in carbonate or faulted rock formations [3].

For more than 60 years, fibre optic sensing (FOS) has been used to measure structural properties, such as integrity and durability, across various industries. In recent years, progress has been made in expanding the abilities of FOS in terms of data and sensing density, expanding even more its fields of application [[4], [5], [6]]. Nevertheless, the usage of fibre optic in rock mechanics testing is still in the early stages. There are two common types of fibre optic sensing systems, dependent on their structure and application: (i) discrete and (ii) distributed sensing. Distributed FOS, such as Brillouin and Rayleigh backscatter systems, measure a fibre's response to strain changes along the entire length of the optical fibre [[7], [8], [9], [10]]. In contrast, discrete FOS measures the strain response of a rock sample at specific locations alongside the sample, similar to conventional strain gauges [11,12]. Fabry-Perot and Fibre Bragg gratings (FBG) sensors are examples of discrete strain sensing [13,14].

Historically, sensing technologies based on Fibre Bragg Grating (FBG) [15] have been applied to a vast range of applications, such as shape sensing [16,17], healthcare [18], magnetic field detection [19,20], as well as in the hydrocarbon industry [[21], [22], [23], [24]]. More recently, the intrinsic properties of the FBG, such as its low electromagnetic interference (EMI), natural waterproofness, multiplexing capability, small size, acoustic emission capabilities, resistance to high pressures and high temperatures (HPHT), wavelength-encoded characters and linearity with pressure [[25], [26], [27], [28]], have been seen as outstanding advantages for using FBG to measure strain, temperature, pressure and ultrasonic waves effectively in a laboratory environment [[29], [30], [31], [32]]. This allows for the obtention of a more accurate and complete picture of the rock behaviour, including all responses derived from the heterogeneity and anisotropy of the sample.

Although a few recent studies explored the possibility of implementing FBG sensor arrays in rock testing, those were limited to unconfined compressive strength (UCS) testing [[32], [33], [34]]. Schmidt-Hattenberger et al. [23] and Sun et al. [35] have successfully used FBG sensors to measure rock deformation/strain in unconfined compressive strength tests under atmospheric conditions. Later, Kovalyshen et al. [36] successfully utilised FBG sensors to measure rock strain under hydrostatic pressure. However, they observed the splitting of FBG peaks as pressure increased, mainly due to the birefringence behaviour of the used fibre.

In this work, we investigated the possibility of implementing Fibre Bragg Grating (FBG) in rock mechanics experiments with and without confining pressure. To distribute FBG sensors at certain locations along the tested samples, we used custom designed and made FBG sensors. Furthermore, we have used a FAZ technology (FAZT) I4 tunable laser based optical interrogator to overcome FBG peak splitting caused by birefringence behaviour during hydrostatic pressure loading, which we experienced in Kovalyshen et al. [36].

**Fig. 1:**
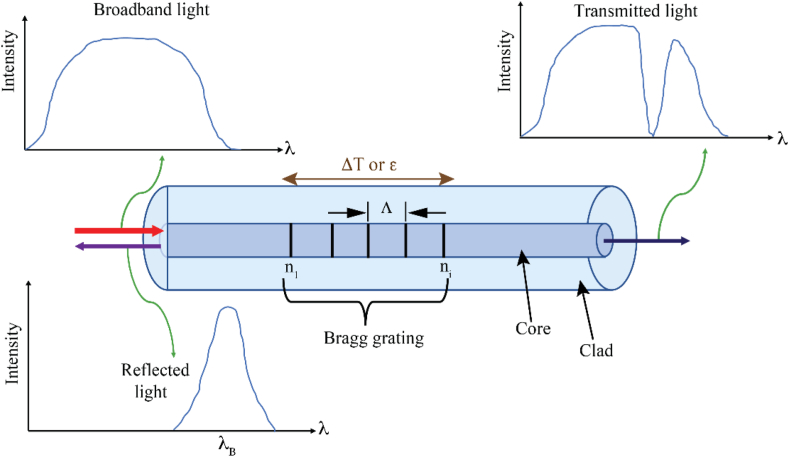
Schematic diagram of the sensing principle of Fibre Bragg Grating [40].

The sensing technology based on fibre Bragg grating (FBG) used in this study works as filters or reflectors with an encoded wavelength that are placed within a short segment of an optical fibre core. When a broadband light is sent into the fibre core, only light with specific narrowband wavelengths will be reflected by the grating, and all other wavelengths will be transmitted, as shown in Fig. 1. The simplistic explanation for the operation of an FBG provides an expression for the peak reflected wavelength (called Bragg wavelength, *λ*_*B*_) as

(1) $$\lambda_{B}=2n\Lambda$$

where n is the effective refractive index of the FBG and Λ is the geometrical grating interval equal to the distance between two successive alterations of the refractive index [[37], [38], [39]].

For the FBG sensors used in this study, the coefficient of the wavelength sensitivity to strain Kε and temperature ($K_{1}$ and $K_{2}$) at room condition are 0.776 p.m./με, 8.53 p.m./^o^C and 0.0023 p.m./^o^C, respectively. The implemented FBG sensors and FAZT I4 were a game changer to deliver long term stability and high precision strain measurements. FBG strain values were compared with conventional strain values obtained by strain gauges.

## 2 Materials and testing methodology

Unconfined compressive strength (UCS) and hydrostatic tests were carried out in Geomechanics and Geophysics laboratory (GGL) of Commonwealth Scientific and Industrial Research Organisation (CSIRO)-Kensington, Western Australia.These tests were conducted to explore and understand the capabilities of the FBG sensing technique in monitoring static strain responses during rock mechanics testing. Standard acrylic (PMMA) and Savonnières limestone (an Upper Jurassic oolitic limestone composed of 99% calcite minerals with a grain size of about 100–200 μm in diameter [41] obtained from a quarry in the North-East of France (Lorraine region [[42], [43], [44]]) were the materials chosen for the laboratory campaign.

One plug made of PMMA and two plugs of Savonnières were prepared following ASTM specifications [45]. Gas porosity and permeability of the rock samples were determined using a porosimeter-permeameter AP-608 (CoreTest Ltd.) under a minimum effective pressure of 250 psi (1.7 MPa). The final plugs dimensions were presented in Table 1.

**Table 1:** Dimensions of the core plugs used for the testing.

Plug type	Diameter (mm)	Length (mm)
acrylic plug	38	76
Limestone plug Sav-3334	38	53
Limestone plug Sav-3330	38	77

The acrylic sample and one of the limestone (Sav-3334) plugs were subjected to unconfined compressive strength (UCS) tests, while the second limestone plug (Sav-3333) was subjected to a hydrostatic test. No pore fluid was used during these tests, and the samples were tested in dry state and at ambient temperature.

One of GGL's universal test machines was used for the UCS tests. It has a load cell capacity of 400 kN and transducers to measure sample axial and radial deformations. The samples were tested according to ASTM [46]. An axisymmetric triaxial vessel from Sanchez Technologies was used for the hydrostatic test. The rig allows for the independent and simultaneous acquisition of radial and axial deformation and stress applied to a cylindrical rock plug. A high precision computer-controlled stepping motor pump was used to control the cell pressure. More information about the rig can be found in Ref. [47].

### 2.1 FBG equipment

In this study, we used custom made FBG sensors with a 125 μm cladding diameter, coated with Ormocer that was provided by FBGS (FBGS Technologies GmbH). We internally designed every single optical fibre to have 8 Bragg gratings, and every grating is a 8 mm long, with a resonant wavelength of around 1550 nm (5 nm difference between two successive FBG sensors on a single wire). The FBG multiplexing capability enables us to select different grating wavelengths for each sensor, with sufficient separation to prevent grating spectra overlapping during testing as stress increases.

**Fig. 2:**
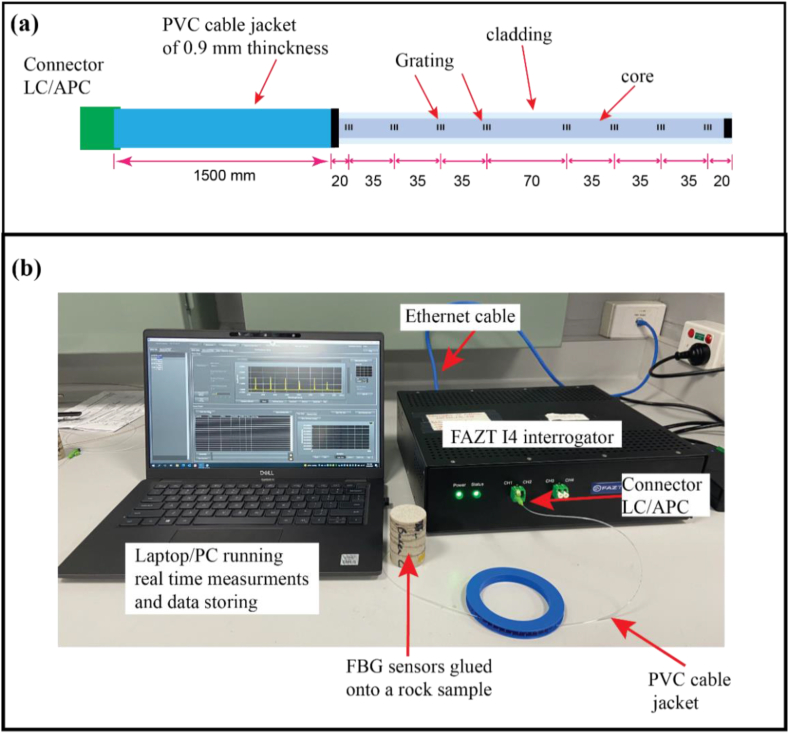
(a) Schematic design of the space between the 8 DTG-FBG sensors used to cover the different sections of the tested samples; (b) setup of the interrogation system used during testing.

The FBG used here was inscribed in a fibre fabrication process known as Draw Tower Grating (DTG), which allows for uniform coating on the grating and improves the tensile strength of the sensor to 5 GPa/breakage strain of >7% [48]. The inscription process causes an undesirable polarisation dependency frequency shift (PDFS) induced by the birefringence in the FBG [25,36,[48], [49], [50]]. As described later, this PDFS needs to be reduced or mitigated during testing. Fig. 2a shows a schematic design of the used distance for the 8 DTG-FBG sensors. The selected DTG-FBG fibre is connected to a 4-channel optical interrogator, the FATZ I4 (Fig. 2b).

The instrument can be operated in full-spectrum and sensor peak detection modes and optimised for static and dynamic measurements. The interrogator returns measurements in 1 p.m. steps across its wavelength range with a sampling rate of 1000 Hz. The system contains photodetectors that detect transmitted and reflected light from FBG sensors with a minimum detectable power (noise floor) at the receiving end of less than −40 dBm. FBG peak processing algorithms can be implemented on a field-programmable gate array (FPGA) that is connected to a computer on board (COB), allowing sensor tracking at a high data rate, as shown in Fig. 2b. The system enables data acquisition/streaming and graphical display software running on a PC via an ethernet connection. Polarisation fluctuations in the fibre path between the interrogator and the sensor need to be isolated, especially for long-term static measurements. This mitigation is achieved using the interrogator's built-in 2-state polarisation switch connecting the laser output and the FBG channels.

### 2.2 Assembly of FBG sensors on samples

There were several conceptual designs on how FBG should be spaced and whereabout would be glued on the sample to cover most of its circumference, without compromising LVDT, conventional strain gauges, radial cantilevers or the experimental work plan. The FBG optical fibre was wrapped around the samples in a helix (radial direction) with circumferential loops along the sample's length. A strong bond between the FBG sensor and the rock sample surface was necessary to achieve the required strain transfer. Thus, several adhesive methods were tested, and Loctite Super Glue All Plastics was chosen. The glue provides adequate coupling between the fibre and sample without altering the local stiffness of the rock [36,42].

For the UCS tests, the end section of the FBG wire was directly connected to the interrogator through an LC/APC connector at the lead-in (Fig. 2). While in the hydrostatic test, the end section was, instead, fed through a pore fluid line, using a special end-platen adapter made for a previous core flooding experiment [36], before being connected to the interrogator.

### 2.3 X-ray CT imaging before and after testing

The Savonnières rock samples were x-ray CT imaged using a Siemens scanner SOMATOM Definition AS at an energy of 140 kV and current of 500 mA. X-ray CT transversal images were acquired with the best field of view capable to achieve a voxel size of 0.1 × 0.1 × 0.1 mm. Avizo 3D software from Thermofisher and Osirix MD software from Pixmeo were used to process the X-ray images and generate 2D and 3D views to observe the samples' inner structure before and after testing. After applying non-local mean filtering to remove some noise from the images, a single threshold on the CT histogram with a CT number less than 500 HU was used to segment the pores/open structures.

A low pass filter was applied to the segmented image to remove small pores and artefacts on the images. In order to only see generated fractures/faults from the geomechanical experiment, an island removal of 50 pixels (i.e., ≤ 5 mm object size) was applied to remove most pores in Savonnieres sample (pore diameter « 5 mm diameter) and keep only the main big size open structures (i.e., >5 mm) like faults [51]. A volume rendering of the segmented images was finally produced on Osirix MD software to visualise the whole structures and match the orientation of the visible structures on the surface of the plug.

### 2.4 Testing procedure

#### 2.4.1 Acrylic sample (PMMA)

**Fig. 3:**
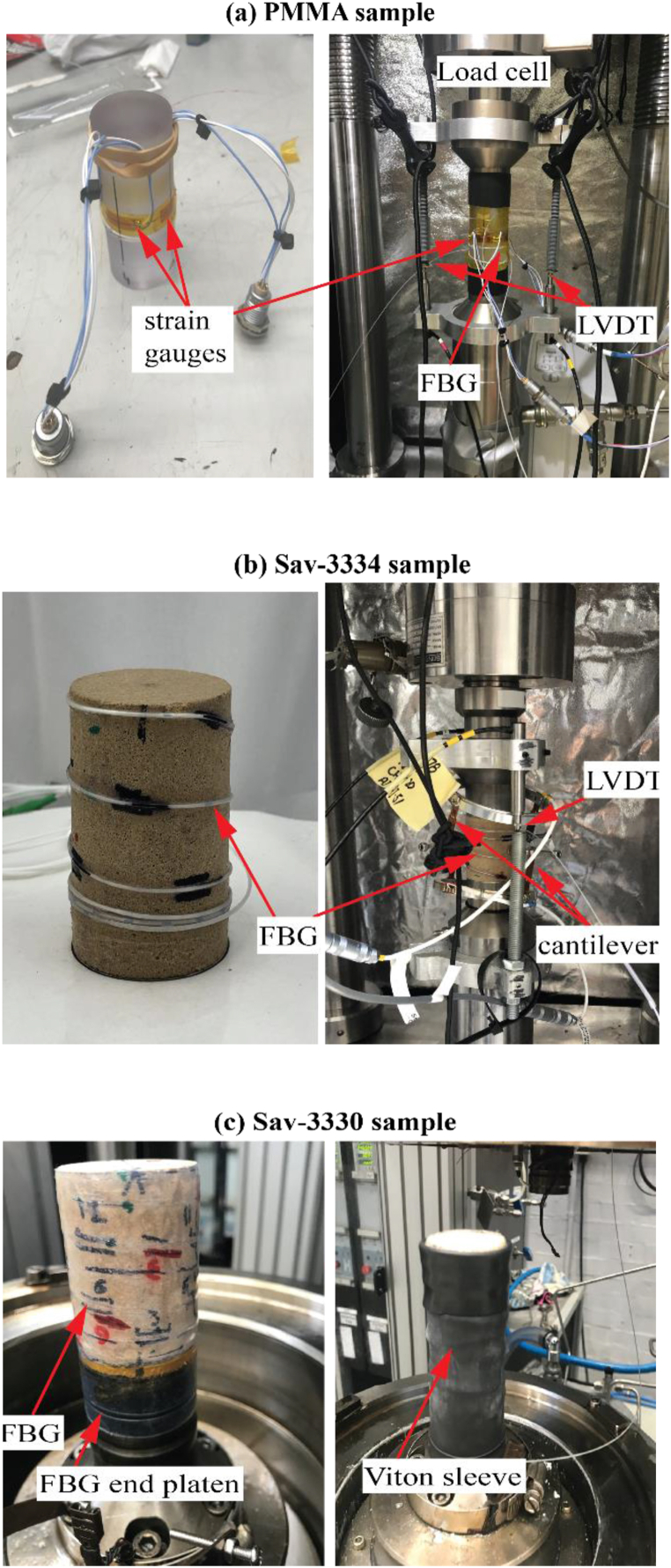
Descriptive photos of the experimental setup for the unconfined compressive strength tests on samples (a) PMMA, (b) Sav-3334 in a uniaxial load frame and for the hydrostatic test on (c) sample Sav-3330 in a triaxial cell.

The radial deformation of the PMMA sample was measured by four FBG sensors and four strain gauges simultaneously to compare the FBG strain data with the conventional strain measurements. Both strain gauges and FBG sensors were glued directly onto the sample surface, at around its mid-height (Fig. 3a). In addition, two diametrically-opposed linear variable differential transformers (LVDT) were clamped on the top and bottom platens to measure axial displacement (Fig. 3a). The sample was axially loaded under a constant average axial displacement rate of 0.259 mm/h until about 5.4 MPa. FBG strains data, radial and axial deformations and axial load were recorded at 1 s. This optimal temporal resolution allowed detailed time-dependent strain-response measurement and manageable data file sizes.

#### 2.4.2 Savonnières samples

• **UCS test on Sav-3334**

**Fig. 4:**
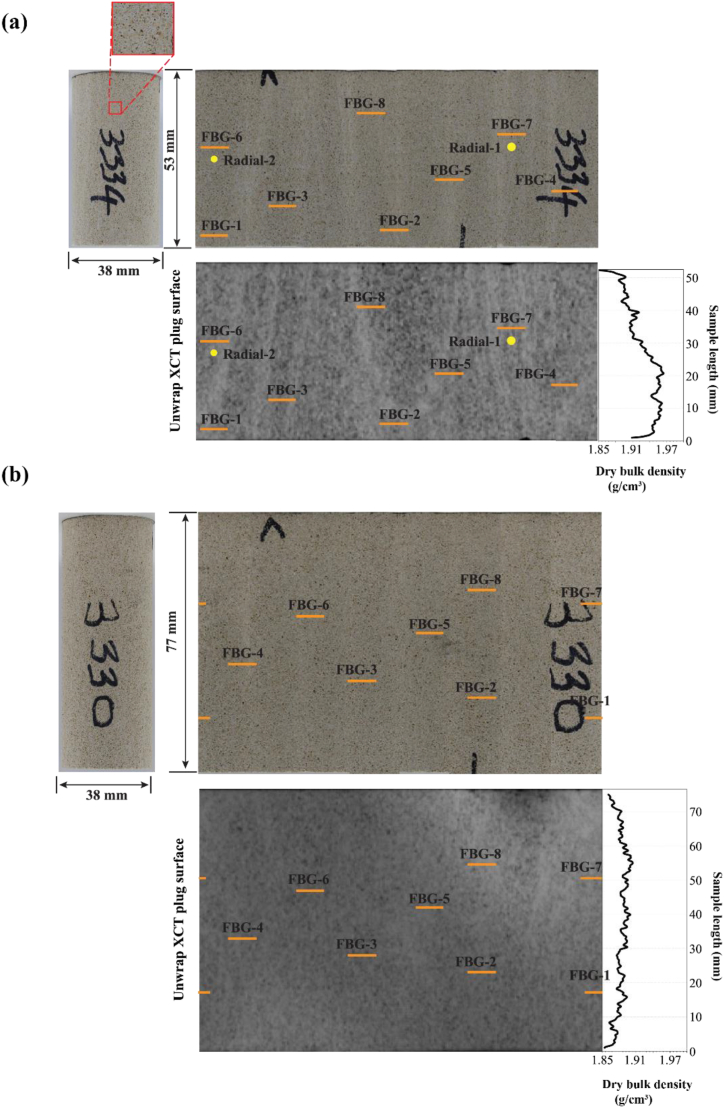
Position of the FBG sensors after unwrapping the plug surface for both Savonnières samples: (a) Sav-3334 used for the UCS test, and (b) Sav-3330 used for the hydrostatic test.

Similar to the PMMA sample, to compare the FBG deformation with conventional strain measurements, for the UCS test on the Savonnières plug, the radial deformation was measured by 8 DTG-FBG sensors (here called FBG-1 to FBG-8) glued onto the plug and two orthogonal cantilever-type radial gauges mounted at the mid-height of the sample, adjacent to FBG-6 and FBG-7 (Fig. 4a). Two LVDTs were also used in this test to measure axial deformation, as shown in Fig. 3b. The sample was axially loaded under a constant average axial displacement rate of 0.259 mm/h until failure. It is worth noting that FBG strain measurements were recorded at a higher sampling rate of 1000 Hz per second and later filtered to 1 Hz/s to match the cantilevers data.

• **Hydrostatic test on Sav-3330**

Another set of 8 DTG-FBG sensors (also labelled FBG-1 to FBG-8) was glued onto the surface of the second Savonnières sample, as shown in Fig. 3c. The location of the FBG sensors on the sample is illustrated in Fig. 4b. To simplify the test, no axial LVDTs, strain gauges or cantilever-type radial gauges were used to measure the sample axial and radial displacements. The sample was inserted into a flexible Viton sleeve and placed inside the pressure chamber (Fig. 3c). The chamber was then filled with hydraulic oil to apply radial/isotropic stress on the sample.

A single feedthrough sealing (rated to 20 MPa and 150 °C) was used to connect the FBG sensors inside the pressure vessel with the interrogator unit through a pore fluid line [42]. The sealing is established by compressing the platen material (PEEK) onto the fibre. The confining pressure was raised at 0.5 MPa/min until the target confining pressure of 20 MPa was reached at an ambient temperature of 23 °C. The sample was then left to equilibrate for a few minutes while the FBG strain changes were monitored and recorded to assess FBG strain reliability during hold off period. Finally, the confining/hydrostatic pressure was unloaded at 0.5 MPa/min.

### 2.5 Analysis procedure

Elastic properties were calculated for all samples based on the available radial and axial displacement readings from the different devices (i.e., strain gauges, cantilevers, LVDTs and FBG sensors). For the UCS tests, Young's modulus (*Ε*) was calculated as the tangential slope of the curve of deviatoric stress versus the average axial strain at 40 to 60% of the yield stress, and Poisson's ratio (ν) was calculated as the tangential slope of each FBG radial strain curve versus the average axial strain for the same stress interval (between 40 and 60% of the yield stress).

For the hydrostatic test, an apparent volumetric strain was calculated based on the radial strain measurements only to understand the volumetric changes undergone by the sample. This volumetric strain was used to graph the correlation of compaction with confining pressure during the loading and unloading stages. It was also used to estimate the sample's bulk modulus, calculated as the ratio of the confining pressure to the apparent volumetric strain described above. For this bulk modulus calculation, we smoothed the confining and the apparent volumetric data by using a moving average with a window of twenty data points. Such modulus is henceforth referred to as apparent bulk modulus to differentiate from the conventional static bulk modulus calculations based on volumetric strain derived from axial and radial strain measurements.

## 3 Results and discussion

Dry gas permeability and porosity for the Savonnières samples were 55 mD and 29%, respectively. X-ray diffraction measurements indicate that the Savonnières material is mostly calcite, with less than 1% quartz [52,53], confirming literature data [41].

### 3.1 Acrylic sample (PMMA)

**Fig. 5:**
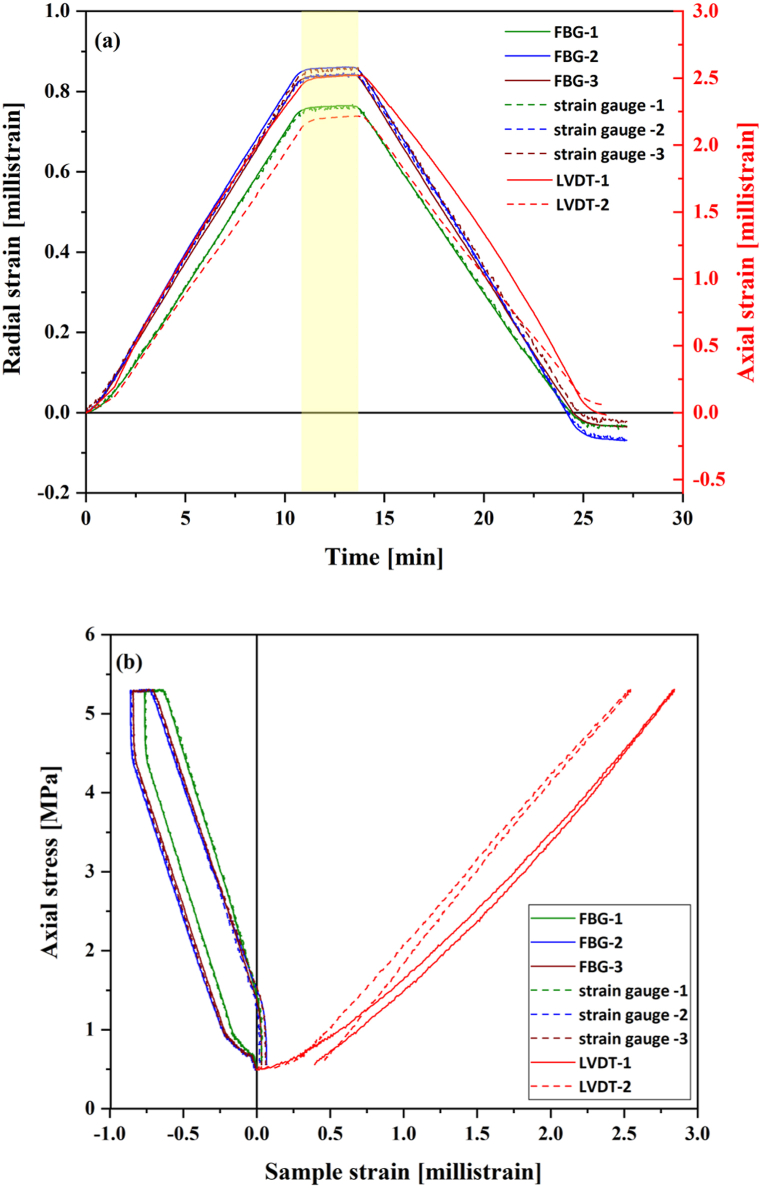
Mechanical results for the PMMA sample: (a) strain vs time and (b) axial stress vs strain. Radial strain from FBG and strain gauges, and axial strain from LVDTs. The yellow rectangular box highlighted the holding period of the axial stress. (For interpretation of the references to colour in this figure legend, the reader is referred to the Web version of this article.)

Both radial and axial strain history for the PMMA sample during the axial loading and unloading cycle, measured by the strain gauges and the LVDTs, are illustrated in Fig. 5a & b. For comparison, the radial strain recorded by FBG sensors was plotted against the one recorded by the radial strain gauges in Fig. 5a & b. Due to unforeseen technical issues with FBG-4 during the test, the results of both strain gauge-4 and FBG-4 were not considered in the analysis. Although the intent was to load the PMMA sample within its elastic strain domain, a slight permanent deformation can be observed.

Nevertheless, as indicated in Fig. 5a & b, the radial FBG strain response is identical to the response of the strain gauges during both loading and unloading stages, which confirms the suitability of the chosen FBG sensors and coupling/gluing technique for further rock mechanics testing. A Young's modulus of 2 GPa was calculated based on the axial strain readings obtained from the LVDT devices. Poisson's ratio values calculated using axial strain data from the LVDTs and radial data from the strain gauges and FBG sensors are presented in Table 2.

**Table 2:** Poisson's ratio values calculated from the UCS test on the acrylic sample.

Sensors	Poisson's ratio	
	based on readings of each sensor	based on the average of all sensors' readings, by sensor type
Strain Gauge-1	0.34	0.34
Strain Gauge-2	0.34	
Strain Gauge-3	0.34	
FBG-1	0.34	0.35
FBG-2	0.35	
FBG-3	0.35	

### 3.2 Savonnières samples

#### 3.2.1 UCS test on Sav-3334

**Fig. 6:**
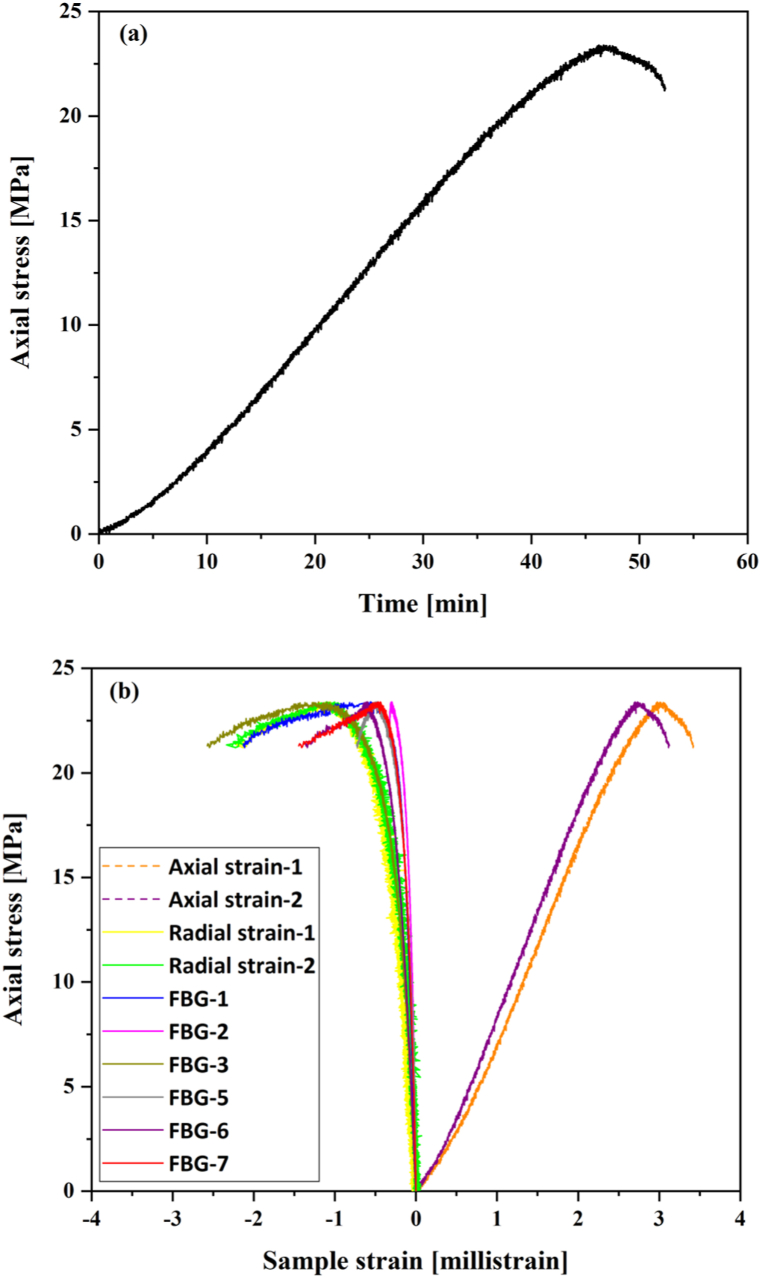
(a) Axial stress until failure vs time, and (b) stress-strain curves including axial strain, FBG radial and two cantilever strain curves of Sav-3334.

**Fig. 7:**
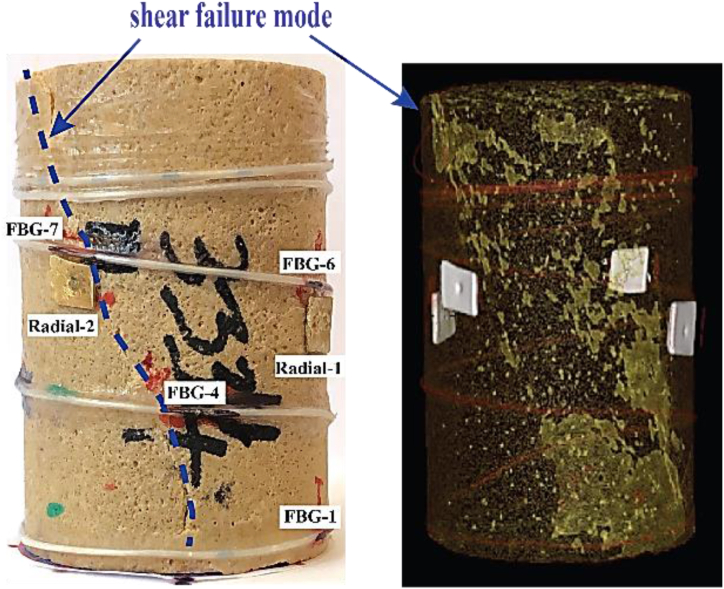
Post-test photo (left) and 3D rendering of the main open structures and segmentation of X-ray CT images of the Sav-3334.

The results of the unconfined compressive test are shown in Fig. 6a & b. The sample reached a peak strength of 23.5 MPa, as shown in Fig. 6a, after which a sharp drop in the load was observed. As previously stated, FBG-6 and FBG-7 are closer to the two orthogonal cantilever-type radial gauges mounted at the mid-height of the sample. Radial strain measurements from the FBG show an excellent agreement with the cantilever-type radial gauges measurements, shown in Fig. 6b. FBG-8 was damaged at an early stage of testing, and thus, it was excluded from the analyses. Additionally, FBG-4 shows different trends from the other FBG sensors at about 10.7 MPa, attributed to the impact of cracks generated before total shear failure occurs near the location of this sensor (shown in Fig. 7). Young's modulus of 14.3 GPa was calculated for this sample. Poisson's ratio values calculated from FBG sensors and cantilever radial gauges are shown in Table 3.

**Table 3:** Poisson's ratio values calculated from UCS test on the Savonnières (Sav-3334) sample.

Sensors	Poisson's ratio	
	based on readings of each sensor	based on the average of all sensors' readings, by sensor type
FBG-1	0.14	0.18
FBG-2	0.11	
FBG-3	0.29	
FBG-5	0.16	
FBG-6	0.23	
FBG-7	0.14	
Radial-1	0.16	0.15
Radial-2	0.15	

XCT images of Sav-3334 after undergoing segmentation of the main structures (see section 2.3) are presented in Fig. 7. A main inclined, slightly twisted shear plane is visible and matches the shear failure seen in the plug's surface image. This failure presents itself throughout the plug as a rough, partially developed surface, making the plug still structurally cohesive (i.e., the shear plane does not completely split the plug into two halves). Some secondary failure planes with vertical strike (along the plug's vertical axis) are present near the top and bottom faces of the plug, but these only propagated about 0.5 cm inside the sample.

#### 3.2.2 Hydrostatic test on Sav-3330

**Fig. 8:**
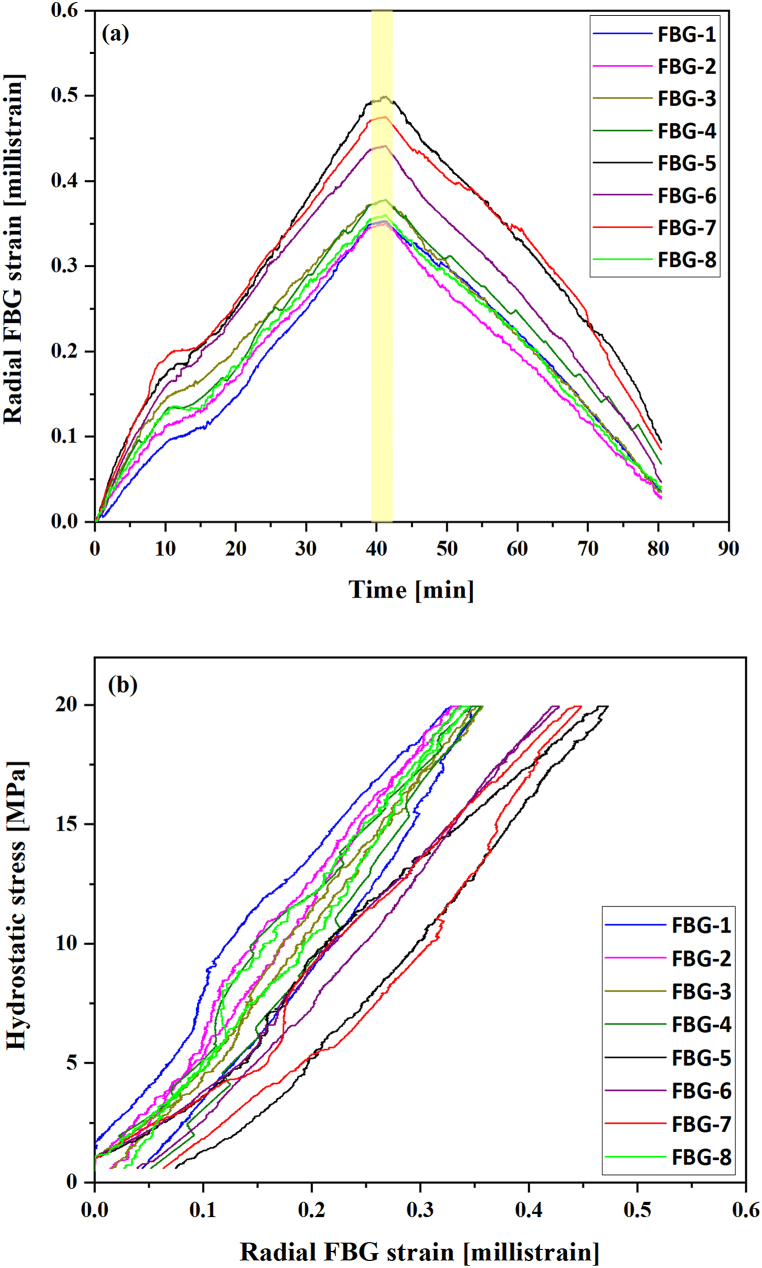
Radial strain data from the eight FBG sensors against (a) time and (b) hydrostatic stress. The yellow rectangular box highlights the holding period of hydrostatic stress. (For interpretation of the references to colour in this figure legend, the reader is referred to the Web version of this article.)

The results of the hydrostatic test are shown in Fig. 8a & b. The maximum applied hydrostatic stress was 20 MPa, shown in Fig. 8b. Cyclic confining pressure yielded inelastic reduction/damage, as FBG strain shows irreversible deformation and relaxation hysteresis during unloading. No significant creep was observed during the holding stage at maximum stress of 20 MPa. By observing the various radial strain curves for each FBG sensor, two prominent trends can be observed: one based on the response of the FBG sensors 1 to 4, and FBG- 8, here called group A, and another trend for the readings of FBG-5, FBG-6 and FBG-7, here called group B.

Variations in the sample's inner structure could be a plausible explanation for the different trends, as well as irregularities on the sample's surfaces, as reported by Bruno et al. [42]. As no striking differences can be observed in the density profile of this particular sample (Fig. 4b), one cannot conclude on correlations between the FBG sensors locations, their strain response and the local density, awarding need for further investigations. On the other hand, the sensor in group B were glued on porous layer sections. Bruno et al. [42] have illustrated FBG sensors response was related to the sample's internal and surface structures.

**Fig. 9:**
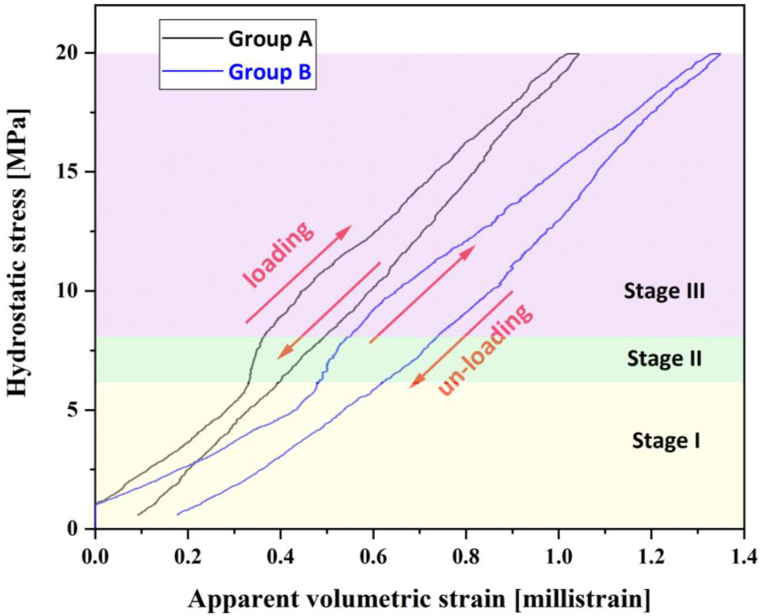
Apparent volumetric strain based on FBG readings during hydrostatic loading and unloading cycle.

Fig. 9 presents the apparent volumetric strain curves based on the average strain results obtained from FBG sensors in groups A and B. Three distinct deformation sections in each volumetric strain loading curve can be observed as follows: Stage I, from the beginning of the test to around 6 MPa; Stage II, from approximately 6 to 8 MPa (a short interval with a slower-paced deformation strike); and Stage III, from approx. 8 MPa till the end of the loading, with a stress-strain trend similar to Stage I, potentially indicating that the Stage II trend was associated with localised deformation only.

**Fig. 10:**
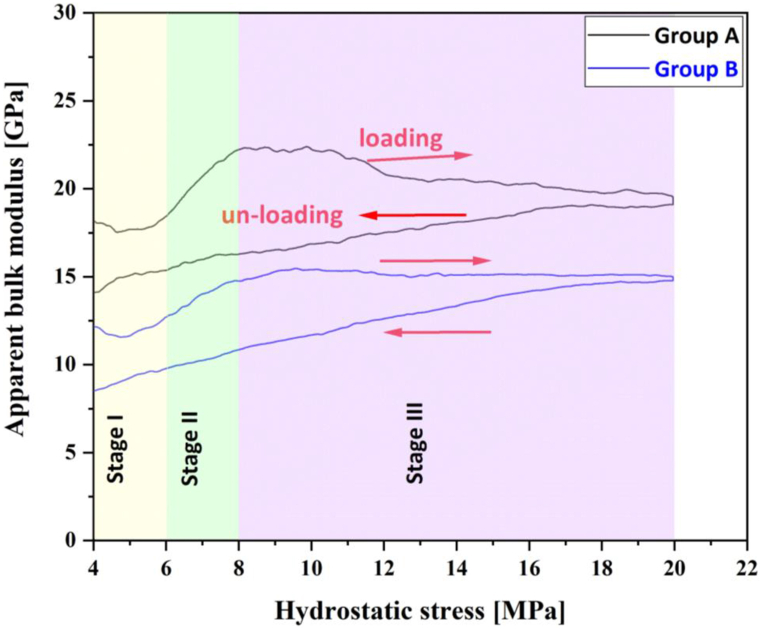
Apparent bulk modulus calculated from volumetric strain based on radial readings from FBG sensors for groups A and B.

Fig. 10 presents the curves of apparent bulk modulus calculated for groups A and B against confining stress. The plot shows that at around 10 MPa confining stress, the apparent bulk modulus reaches a peak of approximately 22 and 15 GPa for groups A and B, respectively, and then stabilises close to those levels until the sample is unloaded. The sample shows uniform behaviour during the unloading, with the apparent bulk modulus reducing steadily and smoothly from the peak value. The occurrence of some level of unrecoverable deformation can also be inferred from the difference between loading and unloading paths.

Table 4 shows the apparent bulk moduli calculated for Stage I (from 1 to 6 MPa confining stress) and Stage III (from 8 to 20 MPa confining stress) for each group, based on the readings of each sensor and also on the average of the reading of all sensors. Apparent bulk modulus values averaged at 14.9 and 10.22 GPa for the two distinct volumetric groups A and B at Stage I, and 17.66 and 14.82 GPa for the same groups at Stage III. Data from Stage II have been disregarded as the stress-strain behaviour of this section seems not to represent the overall behaviour of the sample, as discussed previously.

**Table 4:** Apparent bulk modulus based on readings of FBG groups A and B.

Stage	Group	Sensor	Apparent Bulk Modulus (GPa)	
			Per sensor	Average per Group
Stage I	Group A	Radial Strain from FBG-1	17.19	14.90
		Radial Strain from FBG-2	16.29	
		Radial Strain from FBG-3	12.44	
		Radial Strain from FBG-4	15.11	
		Radial Strain from FBG-8	14.15	
	Group B	Radial Strain from FBG-5	11.01	10.22
		Radial Strain from FBG-6	10.86	
		Radial Strain from FBG-7	8.96	
Stage III	Group A	Radial Strain from FBG-1	16.12	17.66
		Radial Strain from FBG-2	18.19	
		Radial Strain from FBG-3	18.85	
		Radial Strain from FBG-4	16.96	
		Radial Strain from FBG-8	18.32	
	Group B	Radial Strain from FBG-5	13.31	14.82
		Radial Strain from FBG-6	16.36	
		Radial Strain from FBG-7	15.07	

Benchtop (ambient) ultrasonic measurements conducted on this same Savonnières sample in the GGL after the hydrostatic test provided a dynamic bulk modulus value of 16.21 GPa. Although factors such as strain rate, strain amplitude and stress conditions, for example, may impact the comparison between static and dynamic measurements [54,55], it can be said that the dynamic value agrees with the static values obtained for the Stage III of the volumetric deformation, corroborating the more consolidated nature of the sample towards the end of the test. It should also be noted that this sample suffered some minor oil contamination upon removal from the cell after testing.

Similarly, benchtop ultrasonic measurements conducted on another Savonnières sample obtained from the same block as the plug Sav-3330, but never submitted to any mechanical load, yielded a dynamic bulk modulus value of 12.71 GPa. This value is within the range of the lower static values obtained for Stage I (beginning of the test). Lebedev et al. [44] reported on ultrasonic-derived elastic parameters for a Savonnières sample, specifically shear and Young moduli, yielding a dry bulk modulus of 12.2 GPa at ambient conditions. Similarly, Shulakova et al. [56] reported simulated bulk modulus values of around 24 GPa for Savonnières with 29% porosity (the same porosity level as those used in this study).

Regardless of potential dissimilarities in the samples' inner structure and the factors impacting the comparison of dynamic and static moduli, all bulk modulus values mentioned above are similar to those obtained from the FBG readings in this study. Hence, it is here interpreted that the similarity of these values may indicate the accuracy of the FBG readings and that, ultimately, FBG sensors can appropriately measure rock deformation under confining.

## 4 Conclusions

Rock mechanics and rock physics data obtained in the laboratory under controlled stress and temperature conditions are widely used to calibrate field-scale observations of underground rock formations obtained, for example, from wellbore logs and seismic surveys. In this sense, the ability to properly characterise the stress-strain behaviour of both homogenous and heterogeneous materials is crucial to the accuracy of those calibrations. However, testing heterogeneous materials with standard strain measurement devices (strain gauges or radial-cantilever devices) remains challenging. In this study, we explored the possibility of utilising Fibre Bragg Grating (FBG) sensors to measure strain during two distinct rock mechanics experiments: unconfined compressive strength (UCS) and hydrostatic tests. The main findings can be summarised as follows:

I. The strain gauges and FBG sensors yielded identical strain measurements. Consequently, Poisson's ratios calculated using strain data from gauges and FBG showed a good agreement.
II. Estimated static bulk modulus values based on the FBG strain readings (here called apparent bulk modulus) obtained during the hydrostatic test performed on the second Savonnières sample are similar to dynamic values obtained on the same sample and from another Savonnières sample. They were also similar to values reported in the literature. Furthermore, the FBG sensors captured internal structure heterogeneity along the sample length.

In summary, the testing protocol has confirmed the FBG sensing capabilities to record deformation with and without confining and opened horizons to further possibilities that cannot be achieved easily with conventional strain measurement devices, such as 3D strain mapping. However, only three samples, one acrylic PMMA and two limestone samples from Savonnieres were used in this study. For future studies, it is recommended to use several rock samples from different sources, and at different experimental conditions to confirm and ascertain the suitability of FGB to measure deformations.

## Author contribution statement

Bruno da Silva Falcão: Performed the experiments; Analyzed and interpreted the data; Wrote the paper.

Ausama Giwelli: Conceived and designed the experiments; Performed the experiments; Analyzed and interpreted the data; Contributed reagents, materials, analysis tools or data; Wrote the paper.

Melissa Nogueira Kiewiet; Nurudeen Yekeen: Analyzed and interpreted the data; Wrote the paper.

Stephen Banks; George Yabesh; Yevhen Kovalyshen; Ahmed Al-Yaseri; Alireza Keshavarz; Stefan Iglauer: Contributed reagents, materials, analysis tools or data.

Lionel Esteban: Conceived and designed the experiments; Wrote the paper.

Leigh Kiewiet; Ludwig Monmusson: Performed the experiments.

## Data availability statement

Data will be available on request.

## Declaration of competing interest

The authors declare that they have no known competing financial interests or personal relationships that could have appeared to influence the work reported in this paper.
